# Artificial Intelligence in Audiology: A Scoping Review of Current Applications and Future Directions

**DOI:** 10.3390/s24227126

**Published:** 2024-11-06

**Authors:** Andrea Frosolini, Leonardo Franz, Valeria Caragli, Elisabetta Genovese, Cosimo de Filippis, Gino Marioni

**Affiliations:** 1 Maxillofacial Surgery Unit, Department of Medical Biotechnology, S. Maria alle Scotte University Hospital of Siena, 53100 Siena, Italy; andreafrosolini@gmail.com; 2 Phoniatris and Audiology Unit, Department of Neuroscience DNS, University of Padova, 33100 Treviso, Italy; leonardo.franz@unipd.it (L.F.); cosimo.defilippis@unipd.it (C.d.F.); gino.marioni@unipd.it (G.M.); 3 Audiology Program, Otorhinolaryngology Unit, Department of Medical and Surgical Sciences for Children and Adults, University of Modena and Reggio Emilia, 41124 Modena, Italy; valeria.caragli@unimore.it; 4 Audiology Program, Department of Maternal, Child and Adult Medical and Surgical Sciences, University of Modena and Reggio Emilia, 41124 Modena, Italy

**Keywords:** artificial intelligence, audiology, machine learning, diagnostic tools, hearing tests

## Abstract

The integration of artificial intelligence (AI) into medical disciplines is rapidly transforming healthcare delivery, with audiology being no exception. By synthesizing the existing literature, this review seeks to inform clinicians, researchers, and policymakers about the potential and challenges of integrating AI into audiological practice. The PubMed, Cochrane, and Google Scholar databases were searched for articles published in English from 1990 to 2024 with the following query: “(audiology) AND (“artificial intelligence” OR “machine learning” OR “deep learning”)”. The PRISMA extension for scoping reviews (PRISMA-ScR) was followed. The database research yielded 1359 results, and the selection process led to the inclusion of 104 manuscripts. The integration of AI in audiology has evolved significantly over the succeeding decades, with 87.5% of manuscripts published in the last 4 years. Most types of AI were consistently used for specific purposes, such as logistic regression and other statistical machine learning tools (e.g., support vector machine, multilayer perceptron, random forest, deep belief network, decision tree, k-nearest neighbor, or LASSO) for automated audiometry and clinical predictions; convolutional neural networks for radiological image analysis; and large language models for automatic generation of diagnostic reports. Despite the advances in AI technologies, different ethical and professional challenges are still present, underscoring the need for larger, more diverse data collection and bioethics studies in the field of audiology.

## 1. Introduction

The integration of artificial intelligence (AI) into medical disciplines is rapidly transforming healthcare delivery, with audiology being no exception. As audiology traditionally relied on manual diagnostic methods and subjective assessments [1], advances in AI have opened new avenues for enhancing diagnostic accuracy, treatment efficiency, and patient outcomes. AI refers to technologies which enable machines to perform tasks typically requiring human intelligence. The history of AI dates back to the mid-20th century, beginning with the development of strict rule-based algorithm systems in the 1950s and 1960s [2]. The field experienced a significant resurgence in the 2000s due to increased computational power and the advent of big data [3]. Key milestones included the development of machine learning (ML) algorithms (a subset of artificial intelligence which allows systems to learn from data and make predictions or decisions without explicit programming), the rise of deep learning (DL) in the 2010s, which is any type of ML based on artificial neural networks with multiple layers, and more recently the emergence of generative AI and large language models (LLMs) [4]. In fact, AI includes a broad range of technologies, each with distinct capabilities and applications. ML is a fundamental subset of AI which focuses on developing algorithms enabling computers to learn from and make predictions based on data [5]. Unlike traditional programming, which relies on explicit instructions, ML systems enhance their performance through iterative exposure to data [6]. As more data are processed, these systems refine their algorithms to improve their accuracy and efficiency in tasks ranging from pattern recognition to predictive analytics. Within the realm of ML, DL stands out as a powerful technique utilizing artificial neural networks with multiple layers; hence the term “deep” [2]. These networks can model complex patterns in large datasets, making DL particularly successful in image and speech recognition tasks [7]. The ability to automatically learn hierarchical feature representations allows DL to excel in identifying intricate patterns and correlations which might be missed by simpler algorithms. Generative AI represents another innovative branch of AI, involving algorithms designed to create new contents such as text, images, or music by learning from existing data [8]. Techniques such as generative adversarial networks (GANs) and variational autoencoders (VAEs) are central to this field [9]. These methods have shown remarkable potential in applications like medical image synthesis, where they can generate high-quality images for training purposes, and in drug discovery, where they can create novel molecular structures. LLMs like GPT-4 (OpenAI, San Francisco, CA, USA) epitomize the advanced capabilities of AI in understanding and generating human-like text [10]. Trained on extensive corpora of text data, LLMs are adept at tasks such as language translation, summarization, and conversational interaction. They employ DL techniques to process and generate text which is coherent and contextually relevant, making them invaluable tools in fields requiring sophisticated language understanding and generation. Although these models showed promising applications across various medical specialties [11,12], the evaluation of healthcare information provided by generative AI platforms remains a topic of debate and requires careful consideration [13]. In audiology, AI applications range from enhancing the accuracy of hearing assessment to automating hearing aid and cochlear implant (CI) fitting, as well as optimization of auditory rehabilitation processes [14,15,16,17]. The deployment of AI in these areas promises to (1) improve the capabilities of audiologists, (2) reduce the burden of routine tasks, and (3) deliver personalized patient care. Despite the promise of AI, its integration into audiology also raises several challenges. These include data privacy concerns, the need for large datasets for training algorithms, potential biases in AI models, and the requirement of rigorous validation before clinical implementation [18]. Furthermore, many audiologists lack awareness of the potential applications of AI in their field [1]. Addressing these challenges is crucial to realize the full potential of AI in audiology.

This narrative review aims to provide a comprehensive overview of the current state of AI applications in audiology. We will explore the technological advancements and discuss the benefits and limitations of AI in audiology. By synthesizing the existing literature, the review seeks to inform clinicians, researchers, and policymakers about the potential and challenges of integrating AI into audiological practice.

## 2. Materials and Methods

A comprehensive literature search was conducted to identify relevant studies and reviews on the application of AI in audiology. The PubMed, Cochrane, and Google Scholar databases were searched on 5 July 2024 with the following query: “(audiology) AND (“artificial intelligence” OR “machine learning” OR “deep learning”)”. The search was limited to articles published in English from 1990 to 2024 to capture the most recent advances in the field. The PRISMA extension for scoping reviews (PRISMA-ScR) checklist was considered to ensure quality of the review [19]. Articles were selected if they primarily focused on the application of AI in audiology. This investigation included studies which provided empirical data on the effectiveness, accuracy, or utility of AI technologies in the context of audiology practices from diagnosis to treatment. Additionally, reviews and meta-analyses which discussed the integration of AI within the audiology field were critically analyzed, given their comprehensive insights and evaluation of multiple studies. Studies which lacked sufficient data or clear methodology were excluded, as these did not provide reliable or verifiable results.

Data extraction was performed independently by three reviewers (V.C., L.F. and A.F.) to ensure accuracy and comprehensiveness. The information extracted included the study design, AI technology used, audiology application, measured outcomes, and key findings. Discrepancies between reviewers were resolved through discussion and consensus. The extracted data were then analyzed to identify the study design, population, intervention, comparison, and outcome. Studies were categorized based on the type of AI technology (ML, DL, or generative AI), clinical field (hearing and balance), and AI application (e.g., diagnosis and treatment). Qualitative result synthesis was performed.

## 3. Results

**Figure 1:**
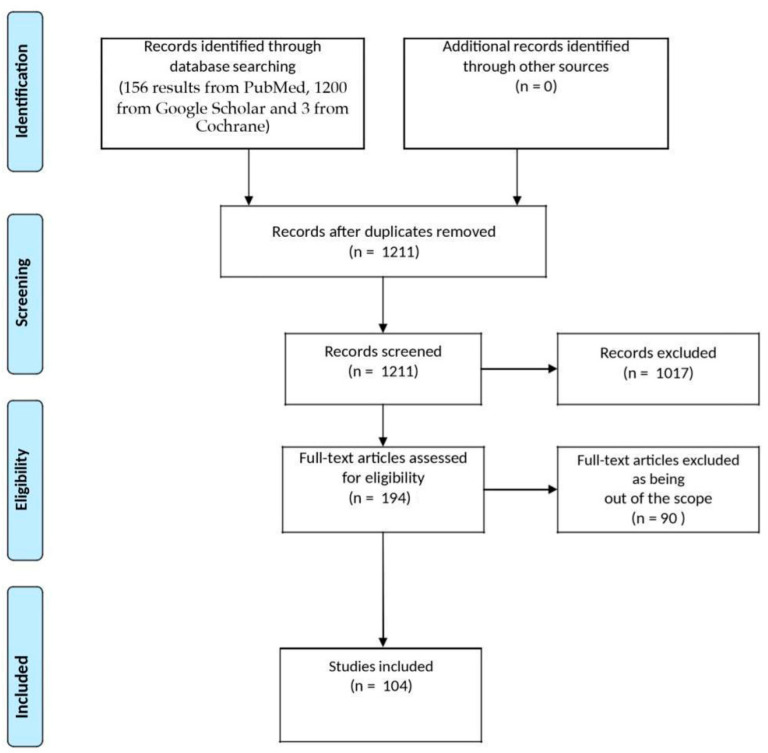
PRISMA diagram of the present review from database search to inclusion.

The database research yielded 156 results from PubMed, 1200 from Google Scholar, and 3 from Cochrane. The selection process led to the inclusion of 104 manuscripts [1,16,20,21,22,23,24,25,26,27,28,29,30,31,32,33,34,35,36,37,38,39,40,41,42,43,44,45,46,47,48,49,50,51,52,53,54,55,56,57,58,59,60,61,62,63,64,65,66,67,68,69,70,71,72,73,74,75,76,77,78,79,80,81,82,83,84,85,86,87,88,89,90,91,92,93,94,95,96,97,98,99,100,101,102,103,104,105,106,107,108,109,110,111,112,113,114,115,116,117,118,119,120,121], as shown in Figure 1.

**Figure 2:**
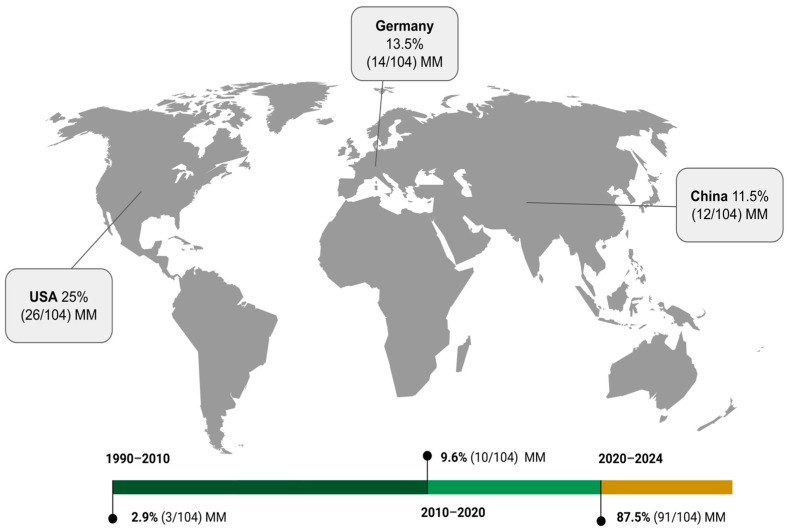
World map depicting top three contributing countries and timeline showing trends of contributions over the last decades. Abbreviation: manuscripts (MM).

The integration of AI in audiology has evolved significantly over the succeeding decades. An early pioneer study by Bonadonna [20] laid the initial groundwork for AI applications in medical audiology, marking the inception of this innovative research area. During the last decade of the second millennium and the first decade of the third millennium (1990–2009), there were only three investigations [20,21,22], representing approximately 2.9% of the total studies reviewed. The second decade of the third millennium (2010–2019) saw the establishment of more sophisticated AI models, improved computational power, and greater data availability. Accordingly, we retrieved a substantial increase, with 10 publications (9.6% of our sample) focused on developing ML algorithms and integrating AI technologies into audiology’s diagnostic and therapeutic tools. The growing trend continued into the third decade (2020–2024), which showed a prolific output of 91 studies from 2020 to 2024, representing approximately 87.5% of the total publications. This period has been characterized by the application of DL, the rise of large language models, and a focus on enhancing diagnostic accuracy, treatment efficiency, and patient outcomes. The three most active countries in this field have been the United States, Germany, and China. The United States contributed to 26 of the reviewed studies, including advancements in hearing tests, diagnostic tools, and therapeutic applications [24,25,29,35,36,37,38,43,45,46,55,60,70,81,82,85,86,96,97,101,104,115,120]. Germany followed with 14 contributions, focusing in particular on ML models and clinical decision support systems [30,33,40,42,51,53,62,63,64,65,73,93,95]. Contributing to 12 studies, China has made significant strides in diagnostic applications using DL and predictive modeling [50,59,78,83,87,105,110,116,117,118,119], as depicted in Figure 2.

Table 1 illustrates the most frequently retrieved journals and the published studies’ designs. Ear and Hearing was the leading journal with 13 publications [24,29,32,35,36,37,50,55,57,69,86,96,97] followed by International Journal of Audiology with 6 [26,30,32,33,42,72], both showcasing significant contributions to the field through various investigations of hearing tests, diagnostics, and therapeutic tools. With four publications, Frontiers in Digital Health reflected the growing multidisciplinary interest in integrating digital health technologies with AI in audiology [43,51,73,115]. Observational studies were the most prevalent study design (68%), followed by development and validation studies (16.3%) and reviews (8.7%).

### Application Fields and Technical Approaches

The application of AI in audiology spans various fields, reflecting the technology’s versatility and potential impact. The most prevalent application was in therapeutic and prognostic tools, accounting for 34.6% of the studies reviewed. These studies focused on using AI to enhance treatment plans and predict patient outcomes, as exemplified by investigations such as those by Jin et al. (2023) and Doborjeh et al. (2023) [84,86]. The hearing test field was close behind, comprising 33.7% of the research, including significant contributions from groups such as Barbour et al. (2019) and Heisey et al. (2020) [29,37], who explored AI-driven methods to improve the accuracy and efficiency of hearing assessments. The miscellaneous category included 21.1% of the studies, covering a range of unique applications and exploratory audiological research areas. The fields of temporal bone radiology and vestibular diagnosis each accounted for 5.8% of the studies. Research in temporal bone radiology, such as the investigation by Masino et al. [25], focused on applying AI to analyze complex oto-radiology imaging data. Meanwhile, studies in vestibular diagnosis, such as those by Kong et al. [87], applied AI to improve diagnosis and treatment of vestibular disorders. Specific features and results of different included categories of investigations will be covered in the following paragraphs.

ML encompasses a diverse array of algorithms, each suited to specific tasks. Choosing the right algorithm is crucial in data analysis and predictive modeling. In the subsequent sections, we explore various representative algorithms, delving into their mechanisms and practical applications in audiology with the aim to enhance understanding of the intricate relationship between function choice and data analysis.

Gaussian process regression (GPR) and Gaussian process classification (GPC) are probabilistic modeling techniques employed for supervised learning and grounded in Gaussian processes (GPs), which are a powerful framework for defining distributions over functions. A GP is essentially a collection of random variables, any finite number of which have a joint Gaussian distribution. This allows GPs to model the uncertainty and variability in the relationship between input and output data in a flexible, non-parametric way [29]. GPR is used for regression tasks, predicting continuous outputs given input data [115]. It models the relationship between inputs and outputs by placing a GP prior over possible functions, leading to predictions which include both a mean estimate and a measure of uncertainty [24]. Instead, GPC is applied to classification tasks, where the outputs are discrete classes. In GPC, the GP models a latent function—an unobserved or hidden function which describes the relationship between the input data and some underlying variable—which is then mapped to class probabilities via a link function, such as a sigmoid function, an unobserved or hidden function which describes the relationship between the input data and some underlying variable [29]. Both approaches benefit from the inherent ability of GPs to quantify uncertainty, making them valuable for tasks where understanding the confidence in predictions is important. Accordingly, they have recently been employed to develop ML techniques for automated hearing threshold tests [24,29].

Unlike standard regression, which minimizes the difference between the predicted and actual values, least absolute shrinkage and selection operator (LASSO) regression adds a penalty term which constrains the coefficient size and promotes sparsity by setting some coefficients to zero [5]. This process selects the most important variables, reduces overfitting, and improves model accuracy and generalizability, making LASSO a valuable tool for creating robust and interpretable predictive models [47]. This technique was encountered in the research line by the Carl von Ossietzky Universität Oldenburg (Oldenburg, Germany) [30,33,40,73]. Specifically, Saak et al. [40] used ML models to predict a cluster of previously defined common audiological functional parameters (CAFPAs) [30]. The CAFPAs—describing functional aspects of the human auditory system (including the hearing threshold, suprathreshold deficits, binaural hearing, neural processing, cognitive components, and socioeconomic status)—were empirically instantiated by an expert survey conducted on a large dataset of audiological measures [33]. Saak et al. [73] had ML models trained on the same dataset, aiming to improve the objectivity and precision of diagnostic decisions by automating the estimation of CAFPAs from patient data. LASSO regression was utilized to identify the most relevant audiological measures influencing specific CAFPAs [73].

The k-nearest neighbors (k-NN) algorithm is intuitive and straightforward, making it suitable for both classification and regression tasks. It classifies new data points based on their similarity to the k-nearest neighbors [122]. In classification, the most common class among the k neighbors determines the new data’s class, while in regression, predictions are based on the average values of these neighbors [50]. Distance metrics such as the Euclidean or Manhattan distance define the term “nearest” [122]. In medical applications, normalization is crucial to ensure variables contribute proportionately to distance calculations, enhancing accuracy and reliability [50]. Choosing the right k value is vital; too small k value can make the model sensitive to noise, while too large k value can oversimplify the model [43]. Although k-NN is easy to implement, it can be sensitive to outliers and computationally intensive for large datasets [50]. The k-NN and artificial neural network (ANN) models were used by Szaleniec et al. [122] to predict hearing improvement after tympanoplasty surgery in patients with chronic suppurative otitis media. The study involved 150 patients, characterized by variables such as age, gender, preoperative audiometric results, ear pathology, and surgical procedure details. The k-NN model, optimized using a 10 fold cross-validation method with the City block distance metric, achieved a prediction accuracy of 75.8% for the validation and test sets. Additionally, the best-performing ANN model demonstrated superior performance, achieving 98% accuracy in the training set, 89% accuracy in the validation set, and 84% accuracy in the test set [122].

A support vector machine (SVM) is used to define the boundaries between data points, treating them as p-dimensional vectors [22]. The goal is to construct a (p-1)-dimensional hyperplane which maximizes the margin from the nearest data vectors, effectively separating different classes [48]. SVMs can handle both classification and regression tasks, making them versatile [25]. SVMs aim to find the global optimal solution, capturing complex relationships within the data. Therefore, they are widely used in medical research. Recently, Rodrigo et al. [45] used the SVM model to predict the outcomes of Internet-based cognitive behavioral therapy (ICBT) for tinnitus. The SVM model, including both linear and radial basis kernel variations, was used to classify treatment success based on a 13-points reduction in the Tinnitus Functional Index (TFI). A study by Rodrigo et al. [45] involved secondary analysis of data from 228 individuals who had completed ICBT with 33 predictor variables, including demographic, tinnitus, hearing-related, and clinical factors. The SVM models were compared with ANN models, and their performance was evaluated using the mean predictive accuracy and area under the receiver’s operating characteristic curve (AUC). Although the ANN model showed the highest predictive accuracy (mean AUC of 0.73), the SVM models also demonstrated adequate discriminative power, with the linear kernel SVM achieving an AUC of 0.72 and the radial basis kernel SVM reaching an AUC of 0.70.

Decision tree (DT) algorithms create hierarchical models with tree-like structures, where nodes represent decision points and branches represent outcomes [28]. This structure is interpretable and systematic, akin to a flowchart [21]. DTs evaluate input features, making decisions at each node to predict the target output. They are versatile, being applicable to both classification and regression tasks [25]. DTs were proposed early to systematically and interpretably classify oto-neurological diseases based on patient symptoms and history, achieving a high accuracy (90% on a database of 564 patients) but facing limitations with insufficient input parameters for certain conditions (e.g., sudden sensorineural hearing loss) [21]. DTs often serve as the basis for ensemble learning methods like random forest (RF) and extreme Gradient Boosting (XGBoost), as discussed below [28].

RF and random forest regression (RFR) combine multiple decision tree algorithms arranged in parallel, enhancing model robustness through ensemble learning. In clinical applications, their use of random inputs and features for each tree results in low correlation between individual trees, effectively capturing diverse patterns in the data [64]. RF and RFR do not require variable normalization, thus accommodating the variability in clinical data [43]. They are valuable for exploring feature importance, and their ability to handle diverse data types, resist overfitting, and provide interpretable insights makes RF and RFR a preferred choice in clinical research and predictive modeling [25]. Hoppe et al. (2022) applied the RFR model to analyze the relationship between age-related hearing loss and speech recognition decline on a large clinical dataset of 19,801 ears. The RFR model provided more specific information about the time course and amount of degradation in speech recognition, yielding smaller mean absolute errors (MAEs) compared with the generalized linear model (GLM). However, the RFR model indicated some degree of overfitting, as evidenced by the differences between the training and test group MAEs. The above-mentioned study found that speech scores varied based on the specific type of hearing loss and the decade of life. Speech recognition deteriorated by up to 25 percentage points over the entire lifespan for constant pure-tone thresholds, with the most significant decline being 10 percentage points per decade [64].

XGBoost constructs an ensemble learning model by adding DTs sequentially. Each new tree corrects the mistakes of its predecessors, creating a highly adaptive model [81]. XGBoost effectively captures complex relationships within data, making it suitable for intricate patterns. However, it is sensitive to variable selection, affecting performance significantly [114]. Despite this, when variables are chosen carefully, XGBoost outperforms other ensemble learning algorithms in predictive accuracy [102]. The investigation by Balan et al. [81] is an appropriate example. The authors applied various ML models to analyze how hearing thresholds could predict speech-in-noise recognition among individuals with normal audiograms. Utilizing archival data of hearing thresholds (0.25–16 kHz) and speech recognition thresholds (SRTs) from 764 participants, XGBoost outperformed the other models—the ANN, deep neural network (DNN) and RF—with an MAE of 1.62 dB. The ANN and RF models showed comparable performances (MAE = 1.68 and 1.67 dB, respectively), while the DNN exhibited poorer performance (MAE = 1.94 dB) [81]. Moreover, Balan et al. [81] highlighted the significant contributions of age and high-frequency thresholds (16 kHz and 12.5 kHz) to the SRT, underscoring the relevance of extended high frequencies in predicting speech-in-noise recognition in individuals with normal hearing.

Inspired by the anatomical model of the human brain, an ANN—more often referred to as just a neural network (NN)—consists of interconnected nodes (the fundamental computational units (i.e., neurons)) which process information through weighted connections (i.e., synapses) [2]. Nodes are categorized into input, hidden, and output layers. Input nodes handle real-world data, hidden nodes process and transform inputs, and output nodes represent predictions [32]. NNs are powerful tools for predictive modeling and ML due to their ability to capture relationships within data [31]. DL, a subset of NNs, incorporates multiple hidden layers, enabling it to handle complex, nonlinear data effectively. DL is particularly adept at processing image data, where convolutional neural networks (CNNs, which are specific DL algorithms able to analyze an input image, assign importance to objects within the image, and differentiate them from one another) are commonly used. CNNs recognize intricate patterns in images, combining features into connected layers for comprehensive analysis. DL’s ability to handle complex datasets has led to its adoption in fields requiring sophisticated analysis techniques [36]. McKearney et al. [32] utilized a deep CNN to classify paired auditory brainstem response (ABR) waveforms into three categories: “clear response”, “inconclusive”, and “response absent”. This innovative approach involved training the CNN on 190 paired ABR waveforms using stratified 10 fold cross-validation and evaluating it on a separate test set of 42 paired waveforms. The CNN architecture included convolutional, pooling, and fully connected layers optimized to detect complex patterns in the ABR data. The network achieved a high classification accuracy of 92.9%, with sensitivity and specificity values of 92.9% and 96.4%, respectively. The study highlighted the potential of DL models to assist clinicians in interpreting ABR waveforms, ultimately improving the consistency and accuracy of hearing threshold estimations [32].

Generative AI refers to algorithms which create new outputs, such as text, images, or music, by learning from existing data, with applications ranging from creative content generation to medical application [108]. Generative AI, particularly large language models (LLMs), represents a significant advancement in AI [1]. LLMs have been trained on vast amounts of text data, enabling them to generate human-like text based on given prompts [11]. These models, such as OpenAI’s GPT-4, have had a disruptive impact and dissemination in the last few years and have shown remarkable capabilities in understanding and generating natural language, making them useful for a wide range of applications [4]. In audiology, the reported use of LLMs encompassed from passing qualification exams to supporting clinical practice and patient education [108,117]. Wang et al. [117] evaluated ChatGPT-4’s performance in the Taiwan Audiologist Qualification Examination. ChatGPT-4 was tasked with answering multiple-choice questions across six subjects, achieving an overall accuracy of 75%, surpassing the passing criterion of 60%. Quite recently, Jedrzejczak et al. [108] longitudinally assessed ChatGPT version 3.5’s effectiveness in providing information on and support for tinnitus. ChatGPT was presented with a set of questions related to tinnitus, and the responses were assessed for accuracy by experts. The study found that ChatGPT’s responses were generally satisfactory and improved over time, but they also identified some limitations, such as the lack of accurate references and occasional misleading information.

## 4. Discussion

The integration of AI in audiology is transforming various aspects of auditory and vestibular health management. In order to systematically explore the advances in these fields, we provided five dedicated subsections of the discussion, focusing on hearing tests, vestibular diagnostics, temporal bone radiology, and therapeutic and prognostic applications.

### 4.1. Hearing Tests

AI is significantly impacting auditory examination practices, particularly in screening, diagnostic, and surgical methods. As traditional gold standard tests for hearing impairment (HI) diagnosis, such as pure-tone hearing tests, are not feasible for large-scale community implementation, AI has been proposed for the development of HI screening text [118]. As a consequence, ML-based HI screening tests could be suitable for the general population and useful for primary care settings [118]. Risk questionnaires have been effectively created using multiple ML algorithms in order to analyze different variables, including patients’ clinical symptoms and hematological test results [118].

To streamline patients’ selection for audiometric evaluation, a separate ML model was also developed to predict the speech-frequency pure-tone average (PTA) based on patients’ demographics, clinical factors, and subjective hearing status. This supervised ML model employed a tree-based architecture to identify individuals needing audiometric testing, thereby optimizing the screening process [85]. For quantitative prediction of hearing thresholds, stimulus-frequency otoacoustic emissions (SFOAEs) elicited by swept tones was also proposed. In this regard, it has been demonstrated that SFOAEs were effective in predicting hearing thresholds and further enhanced through the application of ML models [50]. A stacked ensemble detection method also showed greater performance compared with traditional ABR detection techniques using ML algorithms [69]. It has also been shown that dynamically masked audiograms achieved accurate true threshold estimates and reduced test times compared with current clinical masking procedures [37]. With regard to diagnostic applications, ML employment has primarily focused on the analysis of tympanic membrane images. Advanced DL methods have enabled automatic diagnosis of otitis media based on wideband tympanometry measurements, enhancing the diagnostic capacity [75]. In this context, different studies suggested that AI was able to detect conditions such as tympanic perforations and otitis media and had a significant accuracy score, ranging from 93.67% to 97.9% [59,78].

In terms of treatments, AI has been applied in order to support surgeon indications. In particular, ML was used to assess the effectiveness of Vibrant Soundbridge (VSB) surgery for conductive or mixed hearing loss [47]. Data suggest that ML enhanced the predictive accuracy regarding speech discrimination scores after VSB surgery [47]. AI was also used to predict adult CI appropriate indication using demographic data and standard behavioral audiometry [101]. ML techniques were also used in order to predict the preservation of residual acoustic hearing in patients receiving CIs, enhancing shared clinical decision making and patient outcomes [120]. AI has been used to enhance autonomous CI fitting. In this regard, psychoacoustic self-testing could improve both the fitting process and follow-up care for patients [39]. Moreover, an AI-assisted CI mapping model showed improvements in audiological outcomes, demonstrating comparability or superiority to manual approaches regarding hearing performance and patient comfort [96,97].

### 4.2. Vestibular Diagnostics

**Figure 3:**
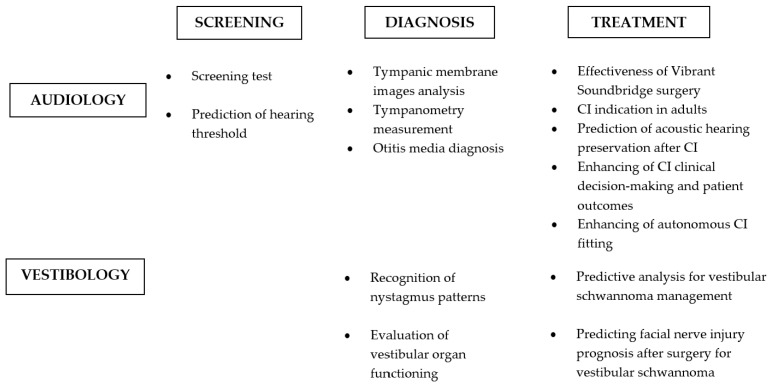
Integration of AI in audiology and vestibology.

AI has also been applied in vestibular disorder assessment. In this regard, AI was used to recognize nystagmus patterns using DL in order to aid physicians in classifying such conditions [87]. Moreover, various ML algorithms were demonstrated to be effective; they achieved an accuracy rate of 94.53% in distinguishing recurrent vertigo types [116]. In order to screen patients earlier, questionnaire-based ML models were also created, predicting common vestibular disorders. Nonetheless, preliminary data suggested the need for objective evidence alongside patient-reported history to improve diagnostic accuracy [99]. Moreover, AI was used to evaluate vestibular organ functioning in the presence of acute or chronic vestibular disorders. In particular, utricular function was assessed in patients with Meniere’s disease, suggesting that AI might be suitable to support diagnosis and target therapies [82]. In terms of treatment, predictive analysis for vestibular schwannoma management showed an accuracy value of approximately 80% using a simple decision tree model [52]. Conversely, Heman-Ackah et al. [106] found 90.5% accuracy in predicting facial nerve injury prognoses after surgery for vestibular schwannoma [106]. Figure 3 reports the current state of the art regarding integration of AI in audiology and vestibology.

### 4.3. Temporal Bone Radiology

#### 4.3.1. AI Models in Temporal Bone Radiology: General Principles

Given the intrinsic complexity of temporal bone anatomy in both physiological and pathological conditions, a comprehensive interpretation of the radiological images of this anatomical district often requires specific training and experience. As a result, the application of AI paradigms to temporal bone radiology represents a rapidly expanding field [123]. Generally speaking, the AI systems applied to the radiology setting start from a wide range of input data (including digital radiological images, clinical data, and demographics) and produce as an output a categorization of the tested cases based on different machine analysis approaches (including supervised learning, unsupervised learning, semi-supervised learning, and reinforcement learning) [124,125]. Supervised learning refers to the use of labeled data (consisting of input features, such as risk factors for specific diseases, clinical data, and demographics paired with corresponding outputs, such as clinical manifestations or diagnosis) in training an ML model [125]. Unsupervised learning involves the use of unlabeled data (with no specific feedback or target variable) to identify patterns of variable distributions, thus allowing tasks such as clustering, dimensionality reduction, and anomaly detection [125,126]. Semi-supervised learning combines elements of both supervised and unsupervised learning (leveraging the smaller amount of labeled data along with a large amount of unlabeled data) to improve the learning process in situations where obtaining labeled data is limited, expensive, or time-consuming [127,128]. Reinforcement learning refers to a trial-and-error training process involving positive or negative feedback in which constant interaction with the environment urges the AI system to choose the appropriate actions to achieve rewarding results [129]. Reinforcement learning approaches include Q-Q-learning, deep Q-Q-networks (DQNs), and policy gradient methods [129].

The applications of AI in temporal bone radiology are mainly based on either supervised or unsupervised learning, with the latter gaining increased interest more recently [123]. The main applications of AI in this field are automated segmentation of temporal bone anatomical structures and assisted diagnosis of middle and inner ear diseases.

#### 4.3.2. Automated Temporal Bone Image Segmentation

Image segmentation refers to the process of partitioning a digital image into multiple segments by identifying and labeling discrete sets of pixels or voxels, representing objects of interest which can be individually analyzed [130]. In temporal bone imaging, the possibility to individually identify anatomical structures, obtaining a quantitative evaluation of their morphological and volumetric parameters, may be valuable support in identifying pathological conditions and anatomical variants [131]. In segmenting the 3D bony structures of the skull, manual and semi-automated tools have long been employed to extract the 3D volume and quantitative information [132]. More recently, the spread of AI-powered technologies has prompted the development of automated segmentation tools specifically for temporal bone anatomic structures [133,134]. The application in medical images of convolutional neural networks specific to image classification and analysis, such as U-Net, allowed the development of automated tools to identify and segment inner and middle ear structures in either RM or CT images with a limited amount of training data [133,134,135]. By analyzing 944 MRI images as a training dataset and 99 for validation, Vaidyanathan et al. [133] aimed to apply U-Net to implement an automatic segmentation system specifically for inner ear structures, including the cochlea and labyrinthic structures, with high performance (true positive rate = 91.5%; false discovery rate = 14.8%; false negative rate = 8.49%). Similarly, Wu et al. [134] and Heutink et al. [135] developed U-Net-powered automated segmentation tools based on CT images specifically for identification of the semicircular canals and cochlea, reporting high accuracy levels (Dice coefficients ≥0.90). Aside from the possibility of in-house development of specific AI-assisted tools, commercially available advanced image analysis software such as Materialise Mimics version 20.0 (Leuven, Belgium: Materialise NV) may also offer the possibility of implementing automated segmentation [131]. When training the Materialise Mimics software with 60 annotated CT scans, Ke et al. [131] reported reliable automatic segmentation of temporal bone structures, obtaining correct identification of facial nerves, ossicles, inner ear (including the cochlea, vestibule, and semicircular canals), internal auditory canal, internal carotid artery, jugular bulb, and external auditory canal in most of the validation images.

#### 4.3.3. AI and Radiological Imaging of Middle Ear Diseases

Moving from technical to clinical aspects, the application of AI models to temporal bone radiology has shown promising results in the field of middle ear disease diagnosis. In this setting, differentiating chronic suppurative otitis media from cholesteatoma is a challenging task, often requiring multi-modal imaging strategies (including both CT and MRI with specific diffusion-weighted sequences) [136,137]. Although still limited to experimental settings, the attempts to employ AI-based image classification models to reduce the need for multiple exams have shown promising results. In 2020, Wang et al. [128] proposed a deep learning framework specifically for the diagnosis of chronic otitis media and cholesteatoma based on temporal bone computed CT scans. A dataset of 975 labeled CT scans was used for training, while the deep learning framework contained two distinct networks: one to extract regions of interest from two-dimensional CT slices and the other to classify images into diagnostic groups based on the extracted regions scans. Such a system showed higher overall accuracy and recall rates in identifying chronic suppurative otitis media and cholesteatoma compared with clinical experts [128]. Using a convolutional neural network architecture trained to perform two consecutive classification tasks on CT images, similar results were found by Chen et al. [138] when discriminating normal versus pathological cases and identifying cholesteatoma versus chronic suppurative otitis media. Such a model showed a substantial gain in identifying cholesteatoma compared with the diagnostic performances of resident fellows and attending otologists [138]. Aside from the automatic image analysis setting, AI has been raising interest as a tool to facilitate the extraction and classification of anatomic and diagnostic information contained in radiology reports. In this intriguing field, Masino et al. [25] proposed an ML system based on natural language processing libraries trained with 726 radiology reports (mainly from temporal bone CTs). In this investigation, they achieved an overall good accuracy in classifying reports by both free-text keywords and ICD-9 terms [25].

#### 4.3.4. AI and Radiological Imaging of Inner Ear

In temporal bone radiology, the need to reliably detect extremely thin anatomical structures on CT and MRI images has raised the issue of optimizing the spatial resolution limit, possibly with no increased image noise. During the last decade, iterative CT reconstruction algorithms allowed the reduction of noise [139]. As a further evolution, the introduction of DL tools has allowed the implementation of more effective image reconstruction algorithms, aiming to obtain substantial noise reduction and, at the same time, improve the spatial resolution [140]. Fujita et al. [141] proposed a deep learning reconstruction algorithm specifically for high-resolution CT images of the temporal bone. They reported a significant reduction in image noise obtained with the deep learning-based reconstruction algorithm, compared with the iterative CT reconstruction approaches. In particular, the depiction of the otic capsule, auditory ossicles, and tympanic membrane was significantly improved in images reconstructed with the deep learning algorithm [141]. Aside from the technical aspects, the possibility to automatedly identify and morphologically classify anatomical structures on temporal bone CT or MRI images, provided by the development of AI-based technologies, has prompted the application of such approaches to the field of inner ear radiology. Ogawa et al. [142] described an unsupervised deep learning system based on a 3D variational autoencoder for detecting and localizing inner ear abnormalities in CT images. They trained their system with unlabeled CT images of a normal subject and used both malformation and normal cases for the test. The unsupervised deep learning system showed good diagnostic performance in identifying cases of malformation, with a specificity of 92% and a sensitivity of around 99% [142].

### 4.4. Therapeutic and Prognostic Tools

During the last decade, accumulating evidence has shown the possible application of AI technologies in supporting clinical decision making by providing clinical experts with probabilities for medical findings or diagnoses which are based on large amounts of patient data [143]. In the field of audiology, the aim of AI-based decision-making support systems is to characterize patients’ clinical profiles, provide presumptive diagnoses, and suggest appropriate treatment or rehabilitation to compensate for functional impairment [62,63]. Such concepts may be applied in clinical practice as the employment of specific AI tools for counseling or self-counseling, therapeutic guidance, and prognostic assessment.

#### 4.4.1. Expert Systems for Counseling and Peer Support in Chronic Audiological Diseases

One of the first applications of AI in clinical management of chronic audiological diseases was the development of expert systems based on inference engines for peer support purposes [23]. In the management of chronic diseases, many forms of peer support have been explored (including person-to-person support, telephone calls, and Internet-based support), with the goal of giving and receiving help founded on the key principles of mutual agreement of what is helpful [144]. AI allows the development of computer-based solutions for self-assessment of symptom severity which can be directly used by patients. Rasku et al. [23] developed an inference engine trained via the collection of necessary and supportive clinical data to profile the severity of Ménière’s disease, as well as the quality-of-life impact in terms of hearing loss, tinnitus, and vertigo. Similarly, Pyykkö et al. [26] reported the impact of an Internet-based, AI-driven peer support program for Ménière’s disease on 740 patients, with 78% of recent onset cases as well as 55% of those with chronic disease rating the program as useful or very useful.

#### 4.4.2. AI-Assisted Therapy and Hearing Rehabilitation

In the audiological field, the application of AI to support therapeutic decisions may be useful, especially in cases in which defining reliable predictive factors of treatment response is intrinsically difficult, such as in patients with tinnitus [45,63]. Regarding this specific setting, in 2021, Schlee et al. [53] proposed some criteria to develop a clinical decision support system specific to tinnitus treatment based on medical, epidemiological, audiological, electrophysiological, genetic, and clinical subtyping data [53]. According to the authors, such a specific clinical decision support system should be able to suggest an optimal treatment strategy for each individual patient, based on the specific input data and in consideration of the previous training dataset [53]. In line with this, Doborjeh et al. [84] proposed the use of a convolutional neural network approach based on electroencephalographic data to identify patients who could most likely benefit from sound therapy. In such an investigation, the prediction accuracy of the convolutional neural network system was 99.07% for the non-responder group and 98.86% for responders [84]. Also, in the field of non-pharmacological approaches to tinnitus, Yin et al. [119] implemented a machine learning model based on the knowledge graph method to identify the likelihood of response of patients with tinnitus to traditional Chinese medicine, based on clinical features and aspects derived from traditional Chinese semiology. According to the authors, such an AI model achieved high prediction performances (99.4% accuracy, 98.5% sensitivity, 99.6% specificity, and 98.7% precision, with an area under the receiver operating characteristic curve of 99%) across 253 test patients [119]. Aside from identification of the predictive factors for treatment purposes in chronic conditions, AI approaches have also been proposed to optimize hearing rehabilitation. In this sense, one of the first applications was AI-based cochlear implant fitting. In 2018, the clinical trial “Programming Cochlear Implant with Artificial Intelligence” [27], based in Belgium, aimed to compare the results in terms of hearing thresholds and speech discrimination of cochlear implants fitted via the AI-powered software FOX^®^ (2G version) with those fitted manually. The first explorative study published from this trial [145] preliminarily showed that patients initially fitted with a traditional approach may experience an improvement in their auditory results when submitted to AI-assisted fitting. In a subsequent study by the same group, a significant improvement in the pure-tone audiometric threshold at 6000 Hz, phonemic discrimination scores, and soft intensity to normal-intensity speech audiometric scores was found after AI-based cochlear implant fitting [96]. The same authors [97] found over a population of 24 patients who received their first cochlear implant, who were randomly assigned to either the manual or FOX^®^-assisted fitting arms, less variability and significantly better speech intelligibility in the experimental group. However, in some cases, the participants reported preferring the manual map because it felt more comfortable, even if the FOX map gave better measured outcomes [97].

#### 4.4.3. AI-Based Prognostic Models

The possibility of managing large data amounts, while also identifying in a hypostasis-free statistical setting the patterns of variable distributions, has led to many AI-based analysis approaches being increasingly employed to identify prognosticators in many medical fields [11,12]. Audiology is no exception among the clinical fields potentially involved in this use of AI. One of the possible applications of AI technology in prognostic assessment regards estimation of the functional hearing correlates of certain clinical conditions. Gathman et al. [85] proposed a machine learning model to predict PTA based on anamnestic data from the patients [85]. Such a system was trained with demographic, medical, and subjectively assessed hearing data labeled with hearing thresholds, and it was able to predict the PTA with a mean absolute error of 5.29 dB [85]. In 2022, Zeng et al. [78] developed a deep learning model based on otoscopic images to estimate the related conductive hearing loss degree. The model was trained on 2232 otoscopic images and validated on 558 images, showing promising performance in predicting conductive hearing loss, with an area under the ROC curve of 0.74 and an accuracy of 81% [78]. In the prediction setting, AI-based models have also been proposed to estimate the functional prognoses of specific diseases. For example, in sudden sensorineural hearing loss, several machine learning models have been proposed to estimate the residual functional outcome, with most of them based on logistic regression, although especially in recent years, other AI-based statistical approaches have been employed, including support vector machines, multilayer perceptron, random forest, deep belief networks, decision tree, k-nearest neighbor, and least absolute shrinkage and selection operator (LASSO) [16]. AI-based predictive models have also been proposed as valuable tools in the prevention and early diagnosis setting. Regarding the prevention of occupational hearing loss, Madahana et al. [67] developed and described a machine learning model based on logistic regression, support vector machine, decision tree and random forest classifier algorithms to classify in real time the levels of noise recorded by sensors worn by mine workers and generate warning reports. Such systems showed good performance in terms of detecting noise exposure, analyzing signals, and generating recommendations to the workers [67]. Finally, many predictive AI-based models have been proposed to estimate the functional yield of cochlear implants [54]. In 2024, Carlson et al. [101] proposed a machine learning model to predict speech discrimination scores after receiving a cochlear implant based on preoperative audiometric and basic demographical data. Such a model achieved an accuracy of 87% in predicting final speech discrimination performances, with a sensitivity of 90% and precision of 80% [101]. Moreover, AI has also been employed to predict the association between brain cross-modal plasticity patterns in deaf patients and the functional outcomes of cochlear implants. In 2021, Kyong et al. [48] tried to predict via a machine learning approach the outcome of cochlear implants based on patients’ brain cross-modal plasticity, measured using event-related responses. In their pilot study, based on the machine learning classification outputs, the authors found that tactile and visual central processing patterns best classified the cochlear implant outcomes, with an accuracy of 98.83 ± 2.57% and 93.50 ± 4.89%, respectively [48].

### 4.5. AI-Driven Augmented Sensors in Audiology

Audiological prostheses, including CIs and HAs, have progressively evolved over the past five decades, transitioning from analog to digital signal processing. The recent integration of AI has further propelled advancements in these technologies, notably improving speech recognition, noise reduction, sound source localization, and the fitting process. These innovations have contributed to significantly better hearing outcomes for users [41]. For HA, AI-driven advancements, such as ML with Bayesian and Gaussian optimization, have allowed personalized real-time fitting parameter adjustments, which improve sound quality in different environments. This is crucial as HAs traditionally rely on average models which do not fully account for individual user needs, despite adequate audiological fitting. An example is Widex’s SSL (WS Audiology, Nymoellevej, Denmark), an AI system integrated into an HA which adapts performance through active patient feedback, improving user satisfaction by optimizing gain and adjusting sound parameters to match its preferences [41]. In a survey on SSL satisfaction, among 118 experienced users, 53 participants had used the functionality. Of those, over 70% reported improvements in at least one listening environment, and 80% would recommend the feature [41]. Speech clarity in noisy environments is one of the primary concerns for HA users. The study by Ting et al. [56] showed how deep learning architectures and data augmentation can improve noise reduction in HAs, specifically through optimizing classification of different ambient noise, leading to better speech clarity in noisy environments [56]. Enhancing the fitting process for HAs and CIs can significantly improve audiologists’ effectiveness, expanding their ability to manage a larger patient population and more effectively addressing the healthcare challenges associated with hearing loss. As discussed in Section 4.4.2, the FOX AI-based fitting systems have proven to be equivalent or more effective than manual fittings while reducing the number of visits required in the first year post operation [96,97]. AI has potential to transform CIs and HAs from traditional prosthetic devices into intelligent augmented sensors. A notable example is the incorporation of inertial sensors into HAs for fall detection and step tracking, as demonstrated by Rahme et al.’s study on the Starkey Livio (Starkey Hearing Technologies, Eden Prairie, MN, USA) [44]. These devices can track steps accurately in real-world and treadmill conditions and detect falls effectively during daily activities, establishing HAs’ role as multi-functional health monitors [44]. Similarly, the “EarGait” system integrated AI and inertial sensors into HAs, allowing for continuous monitoring of gait parameters such as stride time and cadence, with the advantage of bilaterality over traditional gait sensor systems integrated into mobile phones and watches. The system provides valuable insights into the user’s mobility, detecting abnormalities which may indicate fall risk or mobility impairments [95]. The use of CIs as augmented sensors includes their ability to capture valuable electrophysiological data from the cochlear nerve and brain. Real-time analysis of these data can be used to monitor and predict cochlear nerve function and auditory outcomes. In a study by Skidmore et al., ML models were developed to predict the functional status of the cochlear nerve in CI users based on electrically evoked compound-action potential parameters [55]. By analyzing these signals, the model can stratify patients based on their neural function, cochlear nerve dimension, and speech recognition results, offering a way to predict CI outcomes and tailor individual treatment plans. This integration of AI has the potential to improve our understanding of how cochlear nerve health influences auditory processing and long-term CI success. To assist the scientific community in interpreting complex electrophysiological signals, Schuerch et al. [94] provided their intracochlear electrocochleography (ECochG) dataset, which consisted of 4924 signals recorded from 46 ears with CIs during and after surgery. These measures provide insight into cochlear health and neural responses. According to the authors, AI algorithms—particularly deep learning networks—will allow an accurate interpretation of these electrophysiological traces [94]. Thus, AI is transforming CIs and HAs into sensors which not only restore hearing, but also provide real-time data on several different biological parameters. This dual role of CIs as both prosthetic devices and diagnostic tools opens new avenues for hearing function understanding, personalized auditory rehabilitation, and continuous monitoring of auditory health.

## 5. Conclusions

At this moment, a fully comprehensive picture of the state of the art of AI applications in audiology and otology may be hard to achieve, due to the ongoing dramatic evolution of this technology. However, the current review tried to depict an updated summary of the most promising applications of various AI models in this medical discipline, while recognizing the inherent limitations related to the broad inclusion criteria. Judging from this review’s results, a large variety of AI models, based on different algorithms and computational approaches, have been described in the field of audiology. As a result, there are no universally accepted approaches. However, within such a variety, some types of AI models appeared to be consistently used for specific purposes, such as convolutional neural networks for radiological image analysis, large language models for automatic generation of diagnostic reports, and logistic regression or other statistical machine learning tools (e.g., support vector machine, multilayer perceptron, random forest, deep belief network, decision tree, k-nearest neighbor, or LASSO) for clinical predictions. In the field of audiology, despite the promising advances in AI technologies, different challenges, such as the availability of limited datasets and biases inherent in single studies, are still present, underscoring the need for larger and more diverse data collection to enhance predictive capabilities of AI models. Moreover, the ethical and professional concerns which have been raised regarding the application of AI tools in the medical setting should be addressed by specific bioethics studies in the field of audiology. Currently, professional healthcare supervision is essential in every instance where AI is integrated into audiology for patient care.

## Figures and Tables

**Table 1:** Summary of top journals and study designs in AI and audiology research considering manuscripts included in the present review.

Journals	Number of Publications (%)	Study Design	Number of Publications (%)
Ear and Hearing	13 (12.5%)	Observational	71 (68.3%)
International Journal of Audiology	6 (5.8%)	Development and Validation	17 (16.3%)
Frontiers in Digital Health	4 (3.8%)	Reviews	9 (8.7%)
Others	81 (88.7%)	Clinical Trials	3 (2.9%)
		Surveys	2 (1.9%)
		Case Reports and Study Protocols	2 (1.9%)

## Data Availability

The raw data supporting the conclusions of this article will be made available by the authors on request.
